# Supplementary material for: The transcriptional landscape analysis of basal cell carcinomas reveals novel signalling pathways and actionable targets
Source: Life Sci Alliance. 2021 May 10;4(7):e202000651. doi: 10.26508/lsa.202000651 (PMC8200290; doi:10.26508/lsa.202000651)
Supplement: Supplementary file 1 [file LSA-2020-00651_TableS1.docx]

**Supplementary Table 1.** Study characteristics

| Study | Nikolaev | Normal | 26 |
| --- | --- | --- | --- |
|  |  | BCC | 51 |
|  | DeSauvage | Normal | 0 |
|  |  | BCC | 11 |
|  | Atwood | Normal | 8 |
|  |  | BCC | 13 |
| BCC | Types | Nodular | 35 |
|  |  | Superficial | 3 |
|  |  | Morpheaform | 3 |
|  |  | Metatypical | 3 |
|  |  | NA | 31* |
| BCC | Gorlin | Yes | 4 |
|  |  | No | 56 |
|  |  | NA | 15 |
| BCC | Vismodegib | Naïve | 48 |
|  |  | Sensitive | 5 |
|  |  | Resistant | 20 |
|  |  | NA | 2 |
| BCC | Response | CR | 1 |
|  |  | PR | 5 |
|  |  | SD | 5 |
|  |  | NA | 64 |
| BCC | Age | Mean | 74.98 |
|  |  | Median | 76 |
|  |  | SD | 11.23 |
|  |  | n | 49 |
|  |  | NA | 26 |
| Normal | Age | Mean | 76.52 |
|  |  | Median | 77 |
|  |  | SD | 11.67 |
|  |  | n | 25 |
|  |  | NA | 9 |

* Among those, 10 laBCC and 1 mBCC
